# Recent Advances in the Management of EGFR-Mutated Advanced Non-Small Cell Lung Cancer—A Narrative Review

**DOI:** 10.3390/curroncol32080448

**Published:** 2025-08-09

**Authors:** Prabhat Gautam Roy, Davida Reingold, Neha Pathak, Saurav Verma, Aarushi Gupta, Nicholas Meti, Consolacion Molto, Prabhat Singh Malik, Geordie Linford, Abhenil Mittal

**Affiliations:** 1 All India Institute of Medical Sciences, New Delhi 110029, India; prabhat@aiims.edu (P.G.R.); drprabhatsm@aiims.edu (P.S.M.); 2 Department of Radiology, Health Sciences North, Northern Ontario School of Medicine, 41 Ramsey Lake Road, Sudbury, ON P3E 5J1, Canada; dreingold@nosm.ca (D.R.); agupta@hsnsudbury.ca (A.G.); glinford@hsnsudbury.ca (G.L.); 3 Princess Margaret Cancer Centre, Toronto, ON M5G2M9, Canada; neha.pathak@uhn.ca; 4 Versepeeten Family Cancer Center, Western University, London, ON N6A5W9, Canada; saurav.verma@lhsc.on.ca; 5 Department of Medical Oncology, McGill University, Montreal, QC H3A0G4, Canada; nicholas.meti@mcgill.ca; 6 Queen’s Cancer Research Institute, Kingston, ON K7L3N6, Canada; cmolto@lh.ca

**Keywords:** EGFR, Osimertinib, chemotherapy, Amivantamab, Lazertinib

## Abstract

The treatment landscape for EGFR-mutated metastatic non-small cell lung cancer (mNSCLC) has evolved significantly with multiple combination regimens demonstrating superiority over single agent Osimertinib over the past two years. Recent trials such as FLAURA2 and MARIPOSA have explored intensified front-line regimens, with FLAURA2 demonstrating improvement in PFS with the addition of chemotherapy to Osimertinib and MARIPOSA, showing both a PFS and OS benefit with a novel combination regimen of Amivantamab and Lazertinib. However, these regimens are associated with significantly higher toxicity to patients and pose a huge financial and logistical burden to the health care system; therefore, treatment selection must therefore be individualized, considering disease biology, patient fitness, and toxicity burden. Post-progression strategies remain challenging due to resistance mechanisms like EGFR C797S mutations and MET amplification and the lack of data post-progression on novel first-line combinations. Ongoing trials are investigating fourth-generation EGFR TKIs, MET inhibitors, antibody–drug conjugates, and bispecific antibodies in subsequent lines. While regimens like Amivantamab-Lazertinib show promise even in second-line settings, toxicity, cost, and access remain barriers. As therapeutic options expand, biomarker-driven sequencing and personalized care will be critical to optimizing long-term outcomes in EGFR-mutated mNSCLC.

## Simple Summary

EGFR-mutated non-small cell lung cancer (NSCLC) is a common form of lung cancer that is treated with targeted drugs. Osimertinib is commonly used as the first treatment; however, the cancer usually worsens after around 18 months. Therefore, various combinations of targeted therapies with chemotherapy or new drugs to prolong the duration for which cancer stays controlled have been tested. While these newer treatments may help some patients live longer, they also bring more side effects and treatment challenges. It is also unclear whether all patients need combination upfront or it can be reserved for later when cancer worsens. This article reviews the latest treatment options for patients with EGFR-mutated lung cancer, discusses how to select the best first-line treatment, and explores what to do once there is cancer progression after first-line treatment. These findings may help doctors personalize treatment, guide future research, and improve patient outcomes in real-world settings.

## 1. Introduction

Epidermal growth factor receptor (EGFR)-mutated metastatic non-small cell lung cancer (mNSCLC) is the most common oncogene-addicted lung cancer with a variable global prevalence based on ethnicity [1,2]. The molecular landscape of EGFR mutations is diverse, with typical mutations like exon 19 deletion and exon 21 L858R substitution collectively accounting for nearly 90% of all mutations. Atypical alterations include mutations in EGFR like S768I, L861Q, and G719X and exon 20 insertions [3,4].

Following results from pivotal clinical trials, third-generation EGFR tyrosine kinase inhibitors (TKIs) such as Osimertinib have become the standard of care treatment for patients with classical EGFR mutations as they have demonstrated superiority over first- and second-generation EGFR inhibitors [5]. However, median progression-free survival (PFS) on Osimertinib is approximately 18 months. Furthermore, few of these patients have durable remissions and nearly one-third drop out after progression and thus do not receive second-line therapy [5]. Therefore, it is imperative to strengthen the treatment paradigm in first-line treatment.

Recent studies have looked at improving outcomes in first-line treatment. This includes a combination of Osimertinib and chemotherapy in the FLAURA2 trial or alternatively a chemotherapy-free approach in the MARIPOSA trial using a third-generation TKI Lazertinib and Amivantamab, which is a monoclonal bispecific antibody targeting EGFR and c-MET [6,7]. Both these studies have shown promising outcomes with better PFS compared to Osimertinib alone. MARIPOSA also had a positive overall survival (OS) readout recently, with OS for FLAURA-2 showing a similar trend [8].

Despite several new treatment approaches, there is uncertainty regarding the most favorable first-line treatment. This is in part due to excellent tolerability of single agent Osimertinib, but also due to toxicity associated with the addition of chemotherapy and Amivantamab and limited global access to newer drugs. Moreover, none of these strategies are curative. Therefore, appropriate sequencing after progression on first-line therapy constitutes an important clinical challenge. This review will explore the complexities of managing EGFR-mutated mNSCLC in first-line treatment and beyond, in addition to focusing on novel agents and unmet needs in clinical practice. We will also highlight ongoing clinical trials in this space and the need for biomarker-driven research.

## 2. Overview of First-Line Treatment (Table 1)

Osimertinib has been the standard of care for patients with mNSCLC with an EGFR mutation based on the results of the FLAURA study [5]. In this study, Osimertinib was compared to standard of care TKIs as first-line treatment in patients with EGFR-mutated mNSCLC (exon 19 deletions and exon 21 L858R substitutions) (*n* = 556). The results demonstrated superior outcomes with Osimertinib, including significantly longer PFS (18.9 months vs. 10.2 months, hazard ratio [HR] 0.46, 95% confidence interval [CI] 0.37–0.57, *p* value < 0.001), median duration of response [mDOR] (17.2 months vs. 8.5 months, HR 0.39, 95% CI 0.29–0.53, *p* value < 0.001) and OS (38.6 months vs. 31.8 months, HR 0.80, 95% CI 0.64–1.00, *p* value = 0.0046). Grade 3/4 toxicity occurred in 34% of participants in the Osimertinib group versus 45% in the comparator group.

This study also included a significant proportion of patients with central nervous system (CNS) metastases (*n* = 116; 21%). Besides excellent systemic response, Osimertinib also showed a reduction in the incidence of CNS progression, with lower rates of severe toxicity, which made it the preferred first-line therapy option. PFS at 18 months among patients with CNS metastases was 58% (95% CI: 0.40 to 0.72) in the Osimertinib group in contrast to 40% (95% CI: 0.25 to 0.55) in the comparator group (HR, 0.48; 95% CI 0.26 to 0.86); however, long-term CNS-specific results were not reported. Interestingly, patients with exon19 deletion did better with Osimertinib [HR 0.68 (95% CI: 0.051–0.90)] compared to exon 21 L858R substitution [HR 1.00 (95% CI: 0.71–1.40)]. Both males and females seemed to benefit from Osimertinib with no appreciable gender-specific differences in efficacy.

Subsequent to the FLAURA study, the phase III FLAURA2 trial (*n* = 557) evaluated Osimertinib combined with a platinum–pemetrexed doublet (4 cycles) followed by pemetrexed maintenance versus Osimertinib alone in previously untreated EGFR-mutated mNSCLC (exon 19 deletion or exon 21 L858R substitution) [7]. Platinum exposure in the combination arm was typically four cycles (either cisplatin 75 mg/m^2^ or carboplatin AUC 5), and most patients received a median of six additional pemetrexed cycles as maintenance. The trial recruited patients with stable or asymptomatic CNS metastases, which constituted approximately 40% of patients, in each arm. Liver metastases were more frequent in the Osimertinib arm (23.7%) compared to the Osimertinib + chemotherapy arm (15.4%). This combination resulted in an improved PFS for the combination of Osimertinib plus chemotherapy of 25.5 months versus 16.7 months [HR 0.62; 95% CI: 0.49–0.79; *p* < 0.001]. OS data remains immature but second interim OS analysis presented at the World Lung Cancer Conference 2024 (WLCC 2024) showed encouraging early results, with median OS not being reached in the Osimertinib plus chemotherapy arm compared to 36.7 months in the Osimertinib arm (HR 0.75, 0.57–0.97, *p*—0.028) [9]. However, this benefit came at the cost of increased toxicity, with 64% patients experiencing grade 3/4 adverse events (combination arm) compared to 27% (Osimertinib monotherapy arm). In total, 38% of patients had a serious adverse event with 11% discontinuing treatment in the combination arm compared to just 6% in the Osimertinib monotherapy arm [7]. Subgroup analysis from this study revealed that combination therapy performed better in Asian Chinese and Non-Asians compared to Asian Non-Chinese, possibly due to heterogeneity in EGFR mutation subtypes. The differences in response amongst exon 19 deletion and exon 21 L858R substitution, which were noted in the FLAURA study, were not seen in this study. This indicates that the addition of chemotherapy to Osimertinib can offset the poor prognostic impact of exon 21 L858R substitution. Patients with CNS metastasis had a better PFS with the combination therapy compared to Osimertinib monotherapy alone (24.9 months vs. 13.8 months, HR 0.47). Similar benefits were also seen in other high-risk groups including younger patients (HR 0.59), those with liver metastasis (HR 0.63), and those harboring TP53 co-mutations (HR 0.57) [7]. No differences in efficacy were observed based on gender.

A novel chemotherapy-free approach using a newer third-generation EGFR TKI (Lazertinib) and a bispecific EGFR-MET monoclonal antibody Amivantamab was then investigated in the phase III MARIPOSA trial [6]. This study recruited 1074 treatment-naïve patients with EGFR exon 19 deletion or exon 21 L858R substitution and randomized them in a 2:2:1 ratio into receiving Amivantamab-Lazertinib combination, Osimertinib monotherapy, or Lazertinib monotherapy. The results mainly focused on Amivantamab-Lazertinib vs. Osimertinib monotherapy arms. This study included patients with stable or asymptomatic brain metastases (41% in both arms), liver metastases (~15–17% in both arms), and TP53 co-mutation (54% in both arms).

This approach demonstrated an improved PFS of 23.7 months for the combination therapy in contrast to 16.6 months for Osimertinib monotherapy (HR 0.70, 0.58–0.85, *p* < 0.001). The overall response rate (ORR) was similar in both the subgroups at 86% and 85%, respectively. Final OS analysis presented at the European Lung Cancer Conference 2025 (ELCC 2025) demonstrated a statistically significant survival benefit with median OS not being reached for the combination arm compared to 36.7 months in the Osimertinib monotherapy (HR 0.75, 0.61–0.92, *p* < 0.005), 3 year OS (56% vs. 44%) [8]. Notably, cross-over was not permitted in this study, and thus most of these patients in the Osimertinib monotherapy arm did not receive Amivantamab in later lines of therapy.

Toxicity was significant with 75% of patients having a grade 3 or higher adverse event, 49% having a serious adverse event, and 35% discontinuing the combination therapy. These side effects were related to EGFR inhibition (paronychia 69%, rash 64%, diarrhea 32%, stomatitis 30%), MET inhibition (hypoalbuminemia 51% and edema 38%), and other common infusion reactions seen with Amivantamab (65%). Venous thromboembolism was noted in about 40% of patients, necessitating prophylactic anticoagulation. Subgroup analysis revealed that median PFS in patients with exon 19 deletion was 27.9 months (95%CI: 25.1–NE), HR 0.65 (0.51–0.85), which was significantly longer in comparison to patients with exon 21 L858R substitution (24.7 months, 95%CI: 19.5–27.4), HR 0.78, 0.59–1.02). This observation was attributed to exon 19 deletion mutations being more sensitive to TKIs. The addition of Amivantamab was hypothesized to block resistance pathways like MET amplification, which frequently emerge in patients receiving EGFR TKIs. For patients with baseline brain metastasis, the combination showed superiority in median PFS (18.3 months with combination therapy vs. 13 months with Osimertinib monotherapy, HR 0.69, *p* = 0.01). Similarly, benefits were seen consistently in other high-risk groups including patients with liver metastasis (median PFS 18.2 months vs. 11 months, HR 0.58, *p* = 0.017), those harboring TP53 co-mutations (median PFS 18.2 vs. 12.9 months, HR 0.65, *p* = 0.003), age less than 65 years (HR 0.50, 0.39–0.65), and those with detectable circulating tumor DNA (ctDNA) at baseline (20.3 months vs. 14.8 months, HR 0.68, *p* = 0.002) [10]. Both males and females seem to derive equal benefit from this approach.

**Table 1:** Overview of first-line trials in EGFR-mutated mNSCLC.

Trial (Arm)	Median Age (Range)	Liver Metastases	CNS Metastases	TP53 Mutation	Baseline ctDNA	ORR	PFS (Median)	CNS PFS (Median)	OS (Median)
FLAURA—Osimertinib [5]	64 years (26–85)	Not reported	19.0%	Not reported	Not reported	80% (75–85%)	18.9 months (95% CI 15.2–21.4)	CNS PFS (18 months) 58% (95% CI 40–72)	38.6 months (95% CI 34.5–41.8)
FLAURA—Gefitinib/Erlotinib [5]	64 years (35–93)	Not reported	23.0%	Not reported	Not reported	76% (70–81%)	10.2 months (95% CI 9.6–11.1)	CNS PFS (18 months) 40% (95% CI 25–55)	31.8 months (95% CI 26.6–36.0)
FLAURA2—Osimertinib + Chemo [7]	61 years (26–83)	15.4%	41.6%	Not reported	Not reported	83% (78–87%)	25.5 months (95% CI~) (HR 0.62)	24.9 months (patients with baseline CNS mets)	NR (95% CI 38.0- NR), interim HR 0.75, *p*: 0.028
FLAURA2—Osimertinib (monotherapy) [7]	62 years (30–85)	23.7%	39.6%	Not reported	Not reported	76% (70–80%)	16.7 months (95% CI~)	13.8 months (patients with baseline CNS mets)	36.7 months (95% CI 33.2–NR)
MARIPOSA—Amivantamab + Lazertinib [11]	64 years (25–88)	15%	41.4%	56%	69.2%	86% (83–89%)	23.7 months (95% CI 19.1–27.7)	25.4 months (95% CI 20.1–29.5), HR 0.79, *p*: 0.07	NR (95% CI 42.9- NR) (interim HR 0.75, *p* < 0.005)
MARIPOSA—Osimertinib [11]	63 years (28–88)	17%	40%	52.5%	71.4%	85% (81–88%)	16.6 months (95% CI 14.8–18.5)	22.2 months (95%CI, 18.4–26.9)	36.7 months (95% CI 33.4–41.0)

PFS—progression-free survival; ORR—objective response rate; OS—overall survival; ctDNA—circulating tumor DNA.

The first-line treatment of EGFR-mutated mNSCLC has become complex with the arrival of two new treatment options—Osimertinib plus chemotherapy and a chemotherapy-free approach with Amivantamab and Lazertinib. These regimens have shown an improved PFS in large phase III trials and recent data suggests that they also improve OS. However, we believe that there are finer nuances to consider while selecting front-line therapy and all patients may not require a combination approach.

Although most patients on Osimertinib experience chronic grade 1–2 toxicities, the incidence of grade 3–4 toxicities remains low with Osimertinib monotherapy (<5%). Clinical experience suggests that most patients on Osimertinib monotherapy remain functionally adept, enjoy a good quality of life, and require few visits to clinics (minimal time toxicity) [5].

The addition of a platinum doublet adds to hematological (anemia, neutropenia, thrombocytopenia) and non-hematological toxicity (fatigue, nausea) with nearly twice the number of patients discontinuing treatment compared to Osimertinib monotherapy. It also increases the number of hospital visits (three weekly infusions) and lab visits, thereby increasing “time toxicity” [7]. The duration of chemotherapy required to achieve benefits also remains uncertain and whether time-limited chemotherapy can achieve similar benefits as continuous chemotherapy is a relevant future research question given the chronic cumulative toxicities of chemotherapy. It would be interesting to see quality of life (QOL) data from the FLAURA2 study, which would shed light on patient-reported outcomes.

Surprisingly, the chemotherapy-free regimen tested in MARIPOSA trial has even higher rates of adverse events with substantial burden of dermatological and hematological side effects, risk of venous thromboembolism (VTE), and infusion reactions. The rate of discontinuation is nearly three times that of Osimertinib and chemotherapy, thereby highlighting the challenges in administering this regimen. It is also associated with a significant burden on the health care system and an increased amount of time toxicity for patients, as Amivantamab is required to be infused at a weekly interval for five weeks followed by two weekly intervals thereafter [11]. Various strategies have been implemented to try to reduce the associated toxicities including enhanced dermatological prophylaxis [12,13], premedication with dexamethasone for infusion reactions [14,15], and prophylactic anticoagulation, which may need to be incorporated routinely in clinical practice.

Despite these toxicities, there are certain patients who have a high risk of early progression on single agent Osimertinib. These include younger patients (age < 65 years) with brain metastasis at the time of diagnosis, exon 21 L858R substitution, the presence of liver metastasis, and those harboring TP53 co-mutation. The presence of detectable ctDNA and lack of early clearance have also recently been associated with shorter survival [16]. In such patients, the benefits of using a combination therapy may outweigh the risks. For patients without these high-risk features, treatment with single agent Osimertinib remains a very reasonable choice.

Among the different combination regimens available, either the regimen of Osimertinib plus chemotherapy or Amivantamab plus Lazertinib is reasonable given the lack of comparative data between these two. Positive OS benefits seen for Amivantamab and Lazertinib are a step in the right direction; however, the lack of data on patient-reported outcomes from both FLAURA-2 and MARIPOSA makes these discussions with patients challenging. High-risk features including TP53 co-mutation and evaluation of CNS PFS seem more robust in the MARIPOSA trial and this regimen may be preferred in patients with brain metastasis or those with a TP53 co-mutation. However, given the vastly different toxicity profiles of these regimens, a nuanced discussion with patients regarding their goals and preferences is essential. Shared decision making is vital. Data suggest that despite survival benefit, patients may prefer Osimertinib monotherapy over a combination, even at the cost of reduced survival [17]. More studies looking at patient preferences and biomarkers to limit treatment duration and reduce toxicity while benefitting patients at high-risk is imperative.

## 3. Mechanisms of Resistance and Second-Line Treatment

**Figure 1:**
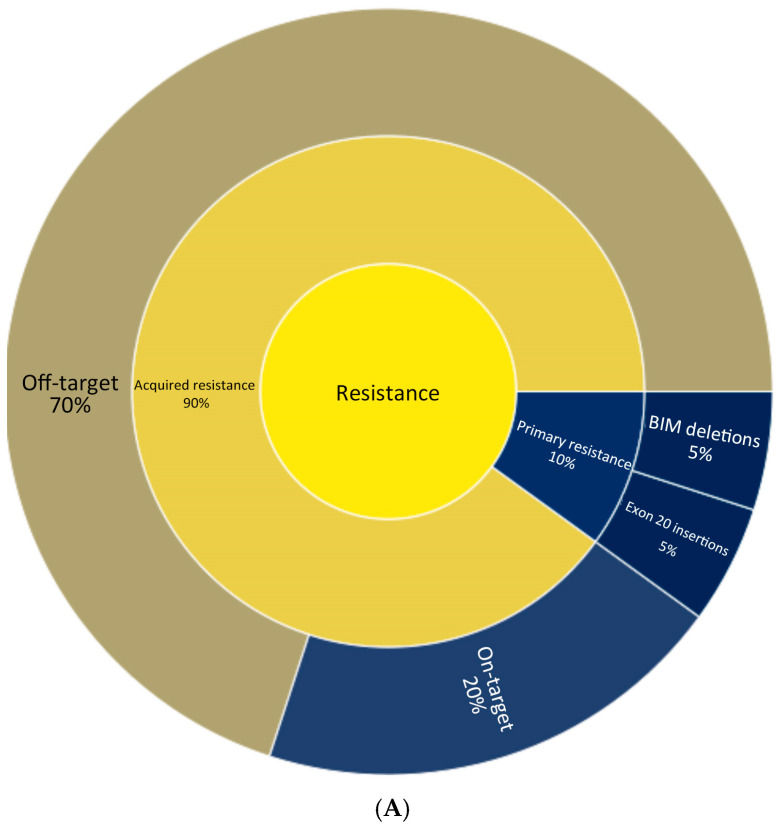

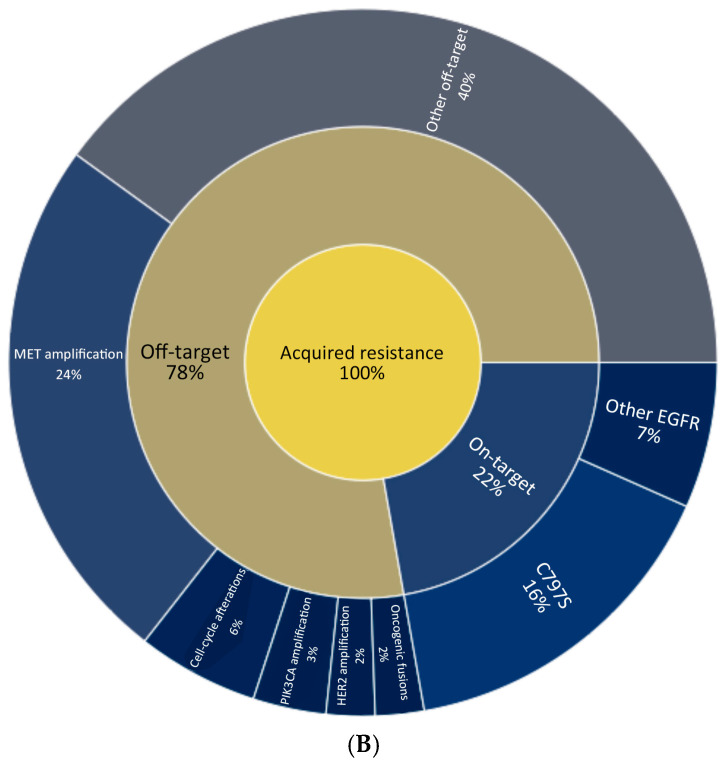
(**A**) Mechanisms of resistance in EGFR-mutated advanced NSCLC [19,20]. (**B**) Mechanisms of acquired resistance in EGFR-mutated advanced NSCLC [21,22].

Resistance to EGFR-targeted therapies can be broadly categorized into primary and acquired resistance (Figure 1A). Primary resistance refers to instances where tumors fail to respond to treatment from the outset. For instance, NSCLCs with specific activating mutations in EGFR, such as exon 20 insertions, are largely unresponsive to most EGFR-TKIs [18]. Additionally, concurrent genetic abnormalities such as BIM (BCL2L11) deletions have been associated with reduced responsiveness to TKIs, even in patients with EGFR-sensitizing mutations [19,20]. In contrast, acquired resistance develops after an initial period of therapeutic efficacy and is classified as either on-target or off-target. On-target resistance mechanisms include mutations in the C797S domain occurring in 14% of cases, while less frequent mutations include C797G, L792H/F, G796S, and L718Q. Off-target resistance stems from mechanisms that bypass EGFR signaling, such as MET amplification (22%), HER2 amplification, PIK3CA amplification, alterations in cell cycle genes, and oncogenic fusions such as FGFR3-TACC3, NTRK1-TPM3, RET-ERC1, and RET-CCDC6 [21,22] (Figure 1B).

In the context of the newer combination strategies discussed above, for patients receiving combination therapy with Osimertinib and chemotherapy in the FLAURA-2 trial, the mechanisms of resistance identified were similar compared to Osimertinib monotherapy; however, fewer patients developed a resistance pathway mutation in the combination arm compared to monotherapy (40% vs. 46%) [23].

Conversely, in the MARIPOSA study, combination therapy with Amivantamab and Lazertinib reduced the emergence of MET amplification (4.4% vs. 13.6% for monotherapy) and EGFR-dependent resistance mechanisms (0.9% vs. 7.9%). There was also a reduction in TP53/RB1 loss (0.9% vs. 2.9%), which has traditionally been associated with transformation to small cell phenotype. Bypass pathway alterations were higher in patients treated with Amivantamab and Lazertinib, including HER2 amplification (7.1%), RAS/RAF kinase mutations (9.7%), PI3K pathway activation (8%), and cell cycle pathway alterations (13.3%). In addition, there was also a higher incidence of complex resistance mechanisms in patients treated with Amivantamab and Lazertinib [24].

## 4. Ongoing Trials and Subsequent Lines of Treatment

Based on available evidence, treatment options for EGFR-mutated mNSCLC in first-line treatment include Osimertinib monotherapy, combination therapy with Osimertinib plus chemotherapy, and a chemotherapy-free combination of Amivantamab and Lazertinib. Post-progression therapy poses a challenge and various strategies targeting different resistance mechanisms are being currently explored in clinical trials (Table 2).

### 4.1. Targeting On-Target EGFR Resistance

Fourth-generation EGFR tyrosine kinase inhibitors (TKIs) are being developed to counter C797S substitution—the prototypical on-target mechanism of resistance emerging after Osimertinib therapy. Early phase I findings with BLU-945, evaluated in the SYMPHONY study, demonstrate favorable tolerability both as monotherapy and in combination with Osimertinib [26]. Additional fourth-generation agents like BDTX-1535 and BBT-176 have been designed to suppress triple-mutant EGFR variants. BDTX-1535, notable for its central nervous system penetration and broad mutant coverage, has produced preliminary tumor regressions in relapsed or refractory EGFR-mutant NSCLC [33]. When T790M and C797S occur in trans, dual inhibition with Osimertinib plus a first-generation TKI restores drug sensitivity. This approach is currently being formally tested in the adaptive ORCHARD platform trial, which includes a cohort receiving Osimertinib + Gefitinib for patients who acquire EGFR C797X after front-line Osimertinib treatment [25].

### 4.2. Off-Target Inhibition

Simultaneous angiogenesis and immune checkpoint blockers have been hypothesized to offset resistance to EGFR-directed therapy based on the ABCP arm of the IMpower150 study. This generated an early signal of efficacy in tumors harboring EGFR or ALK driver alterations [34]. The bispecific antibody Ivonescimab, which co-targets vascular endothelial growth factor (VEGF) and programmed cell death-1 (PD-1), produced encouraging results in a phase II cohort of EGFR-mutant metastatic NSCLC [35]. This has laid the foundation for the HARMONI-A trial, a randomized multicenter study conducted across 55 sites in China with patients who have progressed on prior EGFR-TKI therapy. Ivonescimab combined with platinum-based chemotherapy has prolonged median PFS to 7.1 months versus 4.8 months with chemotherapy alone (hazard ratio 0.46; 95% CI 0.34–0.62). The overall survival results are immature for formal analysis [27].

The MARIPOSA-2 trial tested Amivantamab chemotherapy and Amivantamab-Lazertinib–chemotherapy versus chemotherapy alone in patients who had disease progression on Osimertinib. While this study was not powered to detect differences between arms containing Amivantamab, it showed that either regimen improved PFS compared to chemotherapy alone (median improvement of 2 months for Amivantamab plus chemotherapy and 4 months for Amivantamab-Lazertinib–chemotherapy) [29]. Toxicities were consistent with what was described for Amivantamab and Lazertinib in the MARIPOSA trial; however, OS benefit has not been demonstrated and QOL data is not yet available.

### 4.3. Combined On-Target and Off-Target Inhibition

Multiple strategies have been explored to target MET amplification in conjunction with EGFR inhibition. The MARIPOSA-2 trial included a treatment arm combining Amivantamab, Lazertinib, and chemotherapy; however, the specific contribution of Lazertinib remained unclear as the trial was not statistically powered for this direct comparison [29]. Level 1 evidence supporting the efficacy of subcutaneous (SC) Amivantamab combined with Lazertinib in patients experiencing progression on Osimertinib and chemotherapy was demonstrated in the PALOMA-3 trial. This study highlighted superior tolerability with the SC formulation, reporting reduced infusion-related reactions (13% vs. 66%) and fewer venous thromboembolic events (9% vs. 14%). This study further demonstrated improved clinical outcomes, including a prolonged median PFS with subcutaneous Amivantamab of 6.1 months compared to 4.3 months and an OS benefit (HR 0.62; 95% CI: 0.42–0.92) [36].

Additional therapeutic approaches have examined combining Osimertinib with selective MET inhibitors. The phase Ib TATTON trial (NCT02143466) assessed Osimertinib in combination with Savolitinib, a potent MET inhibitor, in patients with MET-amplified NSCLC who had previously developed resistance following treatment with at least one prior EGFR TKI. This combination achieved an objective response rate (ORR) of 52%, with a median duration of response (mDOR) of 7.1 months [37]. Similarly, the INSIGHT-2 study evaluated Tepotinib, another selective MET inhibitor, in combination with Osimertinib in the same clinical context. This regimen demonstrated an ORR of 50%, mDOR of 8.5 months, median PFS of 5.6 months, and median OS of 17.8 months [31]. In another phase II SAVANNAH study of patients with EGFR-mutated mNSCLC with MET overexpression (3+ by IHC) or amplification (>10 copies by FISH), the combination of Savolitinib plus Osimertinib was superior to Savolitinib alone, again highlighting the importance of continued EGFR inhibition in the presence of MET amplification [38].

### 4.4. Targeting Tumor Antigens—ADC’s

Antibody–drug conjugates (ADCs) have emerged as compelling later-line options as their cytotoxic payloads retain activity across diverse resistance pathways. Datopotamab deruxtecan (Dato-DXd) has received FDA Breakthrough Therapy designation for EGFR-mutated NSCLC that has progressed after Osimertinib and platinum chemotherapy based on the strength of data from the TROPION-Lung05 trial and the EGFR-mutant subset of TROPION-Lung01 [32,39]. In TROPION-Lung05, heavily pre-treated patients achieved an ORR of 43.8% with a median duration of response of approximately seven months. Patritumab deruxtecan (HER3-DXd), an ADC directed against HER3, has likewise demonstrated modest PFS benefit in the phase III HERTHENA-Lung02 study among patients who relapsed after Osimertinib; OS data are still pending [30]. Sacituzumab Tirumotecan (sac-TMT) is a Trop2 directed ADC which has also shown promising clinical activity with an ORR of 45% vs. 16% with docetaxel, median PFS 6.9 vs. 2.8 months, HR 0.30, 0.20–0.46, and 12 month OS 73% vs. 54%, HR 0.36, 0.20–0.66, in a third-line setting in patients who have had disease progression on an EGFR TKI and platinum-based chemotherapy in a randomized trial conducted in China [40].

### 4.5. Currently Available Subsequent Lines of Treatment (Figure 2)

The optimal post-progression strategy is dependent upon the front-line regimen and molecular alterations that subsequently emerge. In patients who relapse after single agent Osimertinib, the combination of Amivantamab plus platinum–pemetrexed chemotherapy is now FDA-approved based on the MARIPOSA-2 study. Whether the addition of Lazertinib meaningfully augments the antibody–chemotherapy doublet remains uncertain, and the triplet is associated with higher toxicity. Moreover, PFS benefit with either regimen is modest at best and neither regimen has yet demonstrated an OS advantage. Subcutaneous Amivantamab promises to be an important step to reduce infusion reactions and mitigate burden on the patients and the health care system; regulatory approval is eagerly awaited. At disease progression, repeat molecular profiling should be pursued to guide enrolment into trials of antibody–drug conjugates or agents matched to specific resistance pathways. Bispecific antibodies such as Ivonescimab are intriguing, but confirmation in larger ethnically diverse cohorts is required prior to widespread adoption. Outside a study context, systemic chemotherapy remains the cornerstone of management. Ablative radiotherapy to oligo-progressive sites can extend first-line benefit; participation in trials such as CURB-2 is encouraged [41,42].

For tumors progressing on the FLAURA-2 regimen (osimertinib plus chemotherapy), Amivantamab combined with Lazertinib is supported by PALOMA-3 data, with the subcutaneous formulation of Amivantamab expected to reduce infusion-related toxicity and improve resource utilization. Given that resistance patterns after Osimertinib + chemotherapy mirror those seen with Osimertinib alone, bispecific antibodies and ADCs are likely to play a central role in this setting as well.

Albeit an OS benefit has been demonstrated with using Amivantamab–Lazertinib in first-line treatment in the MARIPOSA study, its delivery is operationally complex, and no validated options exist once resistance develops. Currently, platinum-based chemotherapy constitutes the standard of care after progression on this doublet and enrolment in clinical trials for drugs matched to resistance mechanisms and ADCs is highly encouraged.

## 5. Conclusions

Targeted therapy has transformed the treatment landscape of EGFR-mutated metastatic NSCLC, with Osimertinib firmly established as the standard first-line agent owing to its excellent systemic and intracranial efficacy, ease of administration, low risk of severe toxicity, and patient preferences for oral therapy. Yet, biologic heterogeneity like the divergent outcomes observed between exon 19 deletions and L858R substitutions demand an increasingly individualized approach. Phase III trials such as FLAURA2 and MARIPOSA, which incorporate platinum–pemetrexed chemotherapy or pairing EGFR inhibition with MET blockade, demonstrate that intensification strategies can extend disease control for biologically high-risk cohorts. However, these gains are counterbalanced by greater toxicity, higher cost, and logistical complexity, thereby underscoring the need for judicious patient selection.

Resistance to Osimertinib is frequently driven by MET amplification or secondary EGFR alterations. Combination regimens that add selective MET TKIs to Osimertinib, alongside fourth-generation EGFR inhibitors capable of suppressing C797S and other tertiary mutations, are emerging as promising salvage options. In the post-progression setting, cytotoxic chemotherapy, antibody–drug conjugates, and clinical trial enrolment based on emerging resistance mutations continue to serve as essential pillars of care.

Moving forward, our aim should be to refine therapeutic sequencing, mitigate cumulative toxicity, and expand access to novel agents. Therefore, advancements in biomarker-directed trial designs that preempt resistance, interventions that delay or forestall disease evolution, and globally inclusive studies to bridge disparities in drug availability are essential for optimal outcome.

## Figures and Tables

**Figure 2:**
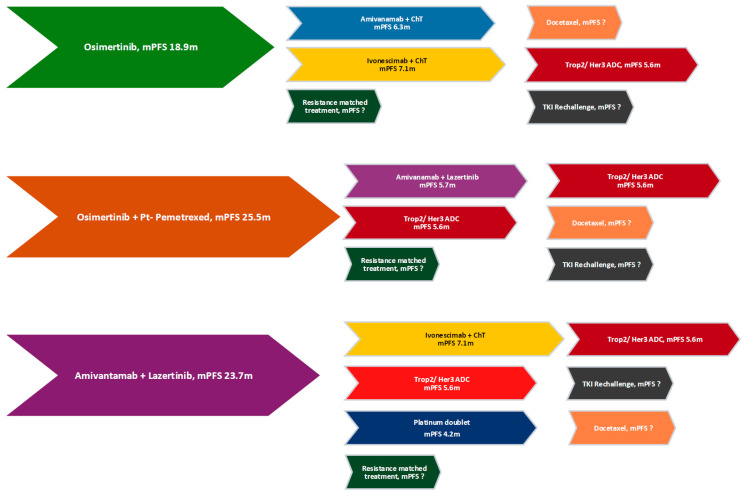
Choosing second-line treatment.

**Table 2:** Ongoing and completed trials in second-line treatment and beyond.

Trial (Phase, NCT)	Phase	NCT	Population (Inclusion)	Intervention(s)	Status	Region(s)	Enrollment
ORCHARD [25]	Phase II	NCT03944772	EGFR—mutant advanced NSCLC with disease progression on first-line Osimertinib	Biomarker-directed multiple arms (e.g., continuing Osimertinib plus Savolitinib, or adding EGFR/HER3-directed ADCs like patritumab-deruxtecan or datopotamab-deruxtecan, cetuximab + gefitinib, etc.)	Active (recruitment complete, treatment ongoing)	Global (multi-continent)	247
BLU-945 SYMPHONY [26]	Phase I/II	NCT04862780	Metastatic EGFR—mutant NSCLC with acquired resistance (e.g., EGFR T790M and/or C797S mutations) after ≥1 prior EGFR TKI	BLU-945 (oral selective EGFR inhibitor)—dose-escalation and expansion cohorts, with and without Osimertinib	Recruiting (ongoing dose-escalation/expansion)	Global (US, Asia, etc.)	(ongoing; not yet reported)
HARMONi-A [27]	Phase III	AK112-301	EGFR—mutant advanced NSCLC progressed on EGFR TKIs (including third-generation Osimertinib)	Arm A: Ivonescimab (anti–PD1/VEGF) Arm B: pemetrexed + carboplatin vs. placebo Arm C: pemetrexed + carboplatin	Completed	China (55 sites)	322
SAVANNAH [28]	Phase II	NCT03778229	EGFR—mutant NSCLC with high MET overexpression and/or amplification, progressed on first-line Osimertinib	Osimertinib + Savolitinib(MET kinase inhibitor)	Recruiting	Global (multicenter)	~360 (enrolled)
MARIPOSA-2 [29]	Phase III	NCT04988295	EGFR—mutant (exon 19del/L858R) advanced NSCLC after progression on Osimertinib	Arm 1: Amivantamab (bispecific EGFR-MET antibody) + platinum chemotherapy (carboplatin + pemetrexed) + Lazertinib; Arm 2: Amivantamab + platinum chemo; Arm 3: Platinum chemo alone	Completed	Global (North America, Europe, Asia, etc.)	657
HERTHENA-Lung02 [30]	Phase III	NCT05338970	EGFR—mutant NSCLC with progression on ≥1 EGFR TKI (including third-gen)	Patritumab-deruxtecan (HER3-directed ADC) vs. platinum doublet (cisplatin or carboplatin + pemetrexed)	Recruiting	Global (Asia, Europe, North America, Oceania)	~560
INSIGHT 2 [31]	Phase II	NCT03940703	EGFR—mutant NSCLC with MET amplification after progression on first-line Osimertinib	Tepotinib (MET inhibitor) 500 mg + Osimertinib80 mg daily	Completed	Multi-national (17 countries)	128
OptiTROP—Lung03 [32]	Phase II	NCT05631262	EGFR—mutant NSCLC after progression on EGFR TKI and platinum-based chemotherapy	Arm A: Sac-TMT Arm B: Docetaxel	Completed	China (48 sites)	137
